# Factors that contribute to social media influence within an Internal Medicine Twitter learning community

**DOI:** 10.12688/f1000research.4283.1

**Published:** 2014-05-29

**Authors:** Tejas Desai, Manish Patwardhan, Hunter Coore

**Affiliations:** 1 Division of Nephrology and Hypertension, East Carolina University – Brody School of Medicine, Greenville, NC 27834, USA; 2 Division of General Internal Medicine, East Carolina University – Brody School of Medicine, Greenville, NC 27834, USA

## Abstract

Medical societies, faculty, and trainees use Twitter to learn from and educate other social media users. These social media communities bring together individuals with various levels of experience. It is not known if experienced individuals are also the most influential members. We hypothesize that participants with the greatest experience would be the most influential members of a Twitter community.

We analyzed the 2013 Association of Program Directors in Internal Medicine Twitter community. We measured the number of tweets authored by each participant and the number of amplified tweets (re-tweets). We developed a multivariate linear regression model to identify any relationship to social media influence, measured by the PageRank.

Faculty (from academic institutions) comprised 19% of the 132 participants in the learning community (p < 0.0001). Faculty authored 49% of all 867 tweets (p < 0.0001). Their tweets were the most likely to be amplified (52%, p < 0.01). Faculty had the greatest influence amongst all participants (mean 1.99, p < 0.0001). Being a faculty member had no predictive effect on influence (β = 0.068, p = 0.6). The only factors that predicted influence (higher PageRank) were the number of tweets authored (p < 0.0001) and number of tweets amplified (p < 0.0001)

The status of “faculty member” did not confer a greater influence. Any participant who was able to author the greatest number of tweets or have more of his/her tweets amplified could wield a greater influence on the participants, regardless of his/her authority.

## Introduction

A number of medical societies, faculty members, and physician-trainees use social media, specifically Twitter, to learn from and educate other social media users ^1, 2^. These social media communities offer a new and exciting medium by which knowledge can be shared and transmitted ^3^. These communities bring together individuals/organizations with various levels of experience ^4, 5^. In the traditional learning model, the learners/students are aware of the authority of the teacher. In Twitter learning communities, however, there are many teachers whose levels of experience can vary. As a result, individuals who participate in Twitter learning communities will be learning from multiple teachers of different levels of experience. This variety can pose a problem because inexperienced individuals can exert a great influence over learners. Although experienced teachers are increasingly participating in Twitter learning communities, whether they are also the most influential members within the learning community is unknown ^3, 4, 6^. We hypothesize that participants with the greatest experience would be the most influential members of one such Twitter learning community.

## Materials and methods

### Data set

We analyzed Twitter messages (tweets) from the 2013 Association of Program Directors in Internal Medicine meeting. This meeting was held from 28 April to 1 May 2013 and brought together residents and chief residents in Internal Medicine with faculty members and program directors. The Alliance for Academic Internal Medicine (AAIM) organized the meeting ( http://www.im.org). The AAIM is a consortium of five academically focused organizations that represent Internal Medicine in the United States: 1) the Association of Professors in Medicine, 2) Association of Program Directors in Internal Medicine, 3) Association of Specialty Professors, 4) Clerkship Directors in Internal Medicine, and 5) Administrators in Internal Medicine. We identified the online Twitter community for this conference through the official hashtag designation established by the AAIM: #APDIM13. Unlike the Twitter learning communities from other scientific meetings, the #APDIM13 hashtag was not created ad-hoc by an unofficial group of conference attendees, but was created and endorsed by the conference organizer (AAIM). Only publicly available tweets and their respective metadata (including author usernames) were collected from the Healthcare Hashtag Project from 28 April to 1 May 2013 ^7^. The Project provides free “firehose” access to researchers who are investigating the use of Twitter at scientific conferences.

### Measuring Twitter activity

We performed two separate analyses to quantify Twitter activity based on the number of tweets authored and tweets amplified. In the first analysis, we categorized tweet authors into one of the following groups: 1) faculty, 2) trainee or residency program representative, 3) organization, or 4) other or unidentifiable. Using metadata, we examined the Twitter profile page of each participant of the Twitter community. We categorized participants as “faculty” if his/her profile page indicated s/he was a faculty member at an academic institution. We identified trainees or residency program representatives if their profile page indicated they were a 1) resident, 2) chief resident, or if the username/profile stated they were a residency program (e.g., @ecuimchiefs). We categorized tweets from the AAIM as “organizer”, as they were all participants that represented a third-party organization. We categorized participants as “other” if the profile page was ambiguous or incomplete. We did not perform an internet search of authors whose Twitter profiles were ambiguous because these profiles were deficient in key pieces of information that would have allowed us to identify them correctly (e.g, absence of full names, absence of photograph, and/or unclear location). Finally, we calculated the number/proportion of tweets per category. The greater the proportion of tweets authored, the greater the Twitter activity.

In the second analysis, we calculated the number of re-tweets per category. Re-tweets are tweets authored by one participant and re-broadcasted (amplified) to a larger Twitter audience by a second participant. We identified re-tweets by the prefix *RT* within a tweet. Participants whose tweets were re-tweeted the most exhibited high Twitter activity.

### Calculating Twitter influence

We measured Twitter influence using Google’s algorithm. The PageRank algorithm quantifies individual influence within an online community ^8– 10^. It assigns a unitless decimal value to each participant based on three factors: 1) the number of times the incident participant is mentioned in the online community, 2) the number of different participants who mention the incident participant and 3) the PageRank of each participant that mentions the incident participant ^11– 13^. For example, if a number of participants mentioned participant A many times, participant A would have a high influence and high PageRank. If a smaller number of participants mentioned participant B, his/her influence would be less than that of participant A ^14^. Each tweet contained the necessary data to determine if the author mentioned another participant.

### Establishing authority amongst participants

We pre-defined “faculty” as individuals from academic institutions who are the most experienced sources of medical information within a Twitter community.

### Statistical considerations

The data set was downloaded and analyzed using Microsoft Excel 2013. We considered Twitter activity measured as number of tweets and number of tweets re-tweeted, as a continuous variable. We used NodeXL to calculate PageRank as a continuous variable. We used the Fruchterman-Reingold algorithm to develop a directed network map of influence ^15^. Nominal variables included each of the four categories assigned to a participant (faculty, trainee, organization, other/unknown). We used the Chi-square test to compare the nominal variables; t-tests and ANOVA for continuous variable comparisons. We developed a multivariate linear regression model, based on standard least squares, to identify the factors that predicted online influence. JMP Pro 10.1 was used to perform all statistical analyses. We performed a word frequency analysis using NVivo 10. This investigation was exempt from review by the Institutional Review Board because the data set is part of the public domain according to Section 102 of the United States Copyright Act ^16^. To the best of our knowledge, this investigation conforms to STROBE guidelines for observational research and SAMPL guidelines for statistical reporting ^17, 18^.

## Results

**Figure 1.**
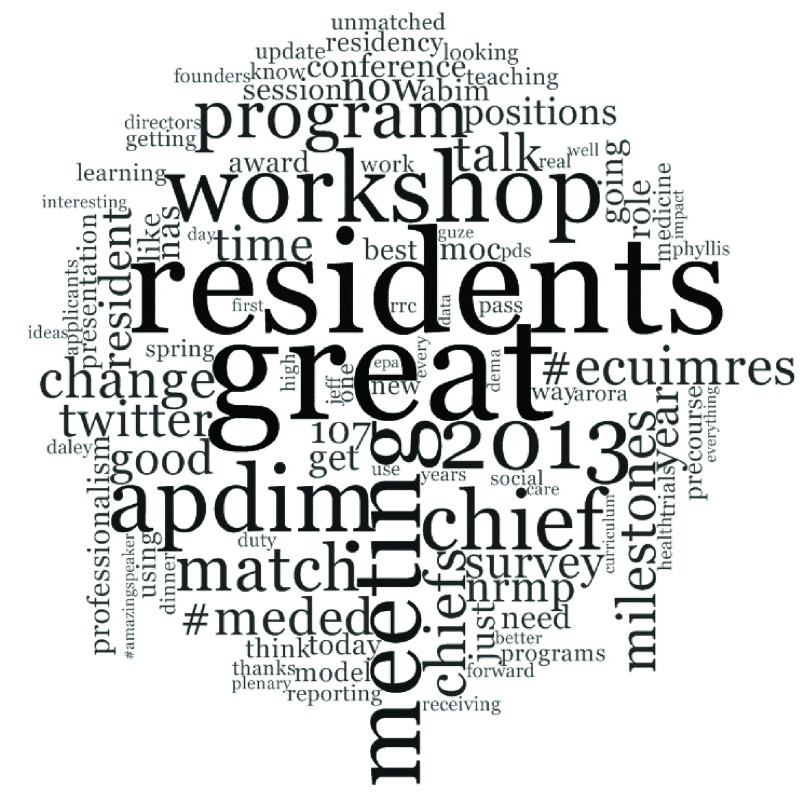
Word cloud of tweets. The size of a word represents its relative frequency within the dataset. Word cloud excludes conjunctions, prepositions, articles, specific Twitter usernames (@username), and #APDIM13.

One hundred thirty two participants authored a total of 867 tweets. Common words used in these tweets included: “great”, “residents”, and “meeting” ( Figure 1). We identified less than two of every ten participants as a faculty member based on the information from their Twitter profile (19%, 95% CI 13–26%, p < 0.0001). However, the faculty members authored approximately half of all tweets (49%, 95% CI 46–53%, p < 0.0001). Six of every 10 participants did not provide enough information on their Twitter profile to be categorized. There were 261 tweets that were re-tweeted (amplified). Faculty members authored the largest number of amplified tweets (52%, 95% CI 46–58%, p < 0.0001) ( Table 1).

**Table 1.** Baseline characteristics.

	Participants		Number of all tweets		Number of tweets re-tweeted	
Participant category	N	% of Total	N	% of Total	N	% of Total
Trainee or residency program	19	14%	135	16%	42	16%
Faculty	25	19%	429	49%	135	52%
Organization	11	8%	101	12%	34	13%
Other or cannot categorize	77	58%	202	23%	50	19%

**Figure 2.**
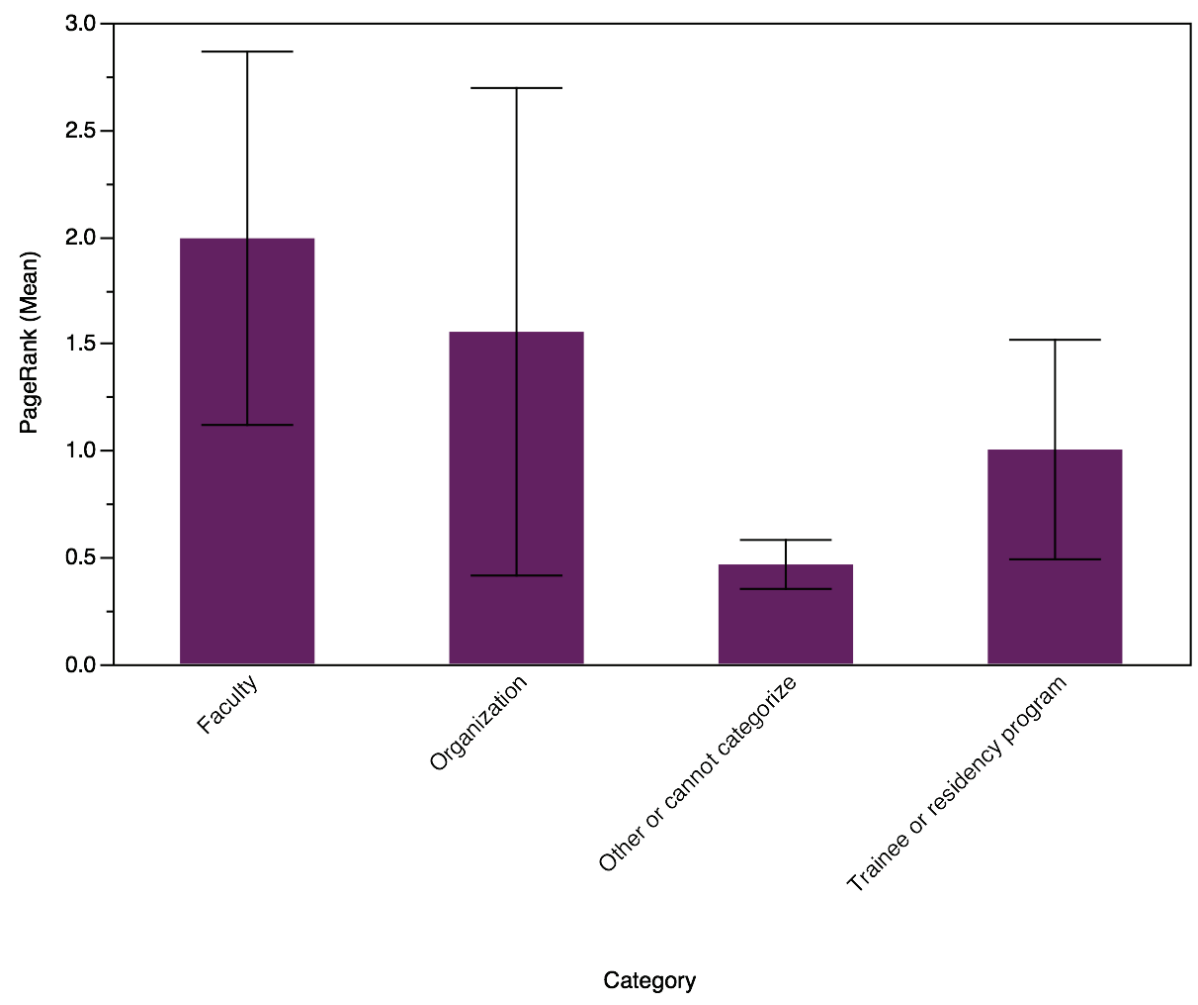
Mean PageRank by participant category. Error bars represent 95% confidence intervals.

**Figure 3.**
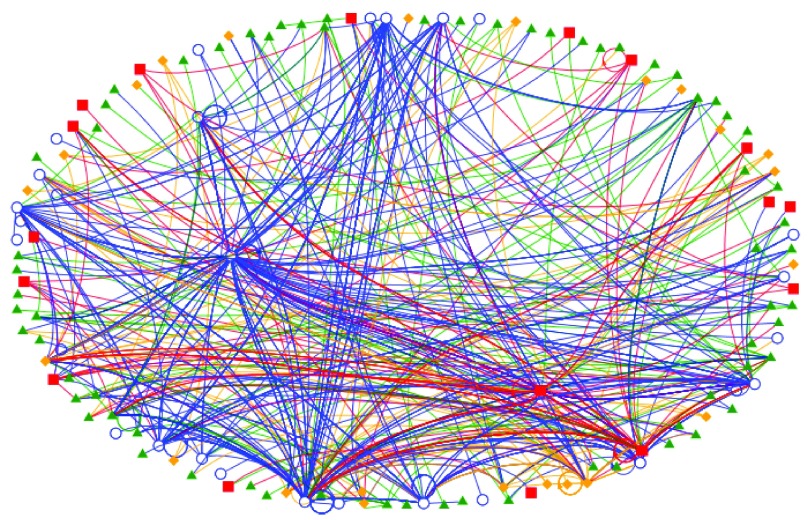
Directed influence map of the #APDIM13 Twitter community. Green triangles (and edges) represent an uncategorized participant, blue circles/edges represent a faculty member, red squares/edges represent organizations and orange diamonds/edges represent trainees or residency programs. Edges are weighted equally. Arrowheads denote the participant who is being mentioned.

The mean PageRank for all participants was 0.92 (SD 1.31). Faculty members had the greatest mean PageRank of 1.99 (95% CI 1.53–2.46). This PageRank was statistically greater than that for trainees (1.00, 95% CI 0.47–1.54, p 0.007) and those participants who could not be categorized (0.47, 95% CI 0.20–0.73, p < 0.0001) ( Figure 2). Figure 3 shows a pictorial representation of the influence exerted by each participant. The map shows that participants identified as faculty had the largest number of mentions (large density of blue circles/edges).

We developed the following multivariate linear regression model:

PageRank = 0.51 + 0.061*(number of tweets authored) + 0.067*(number of tweets re-tweeted) – 0.25*(1 if category = other) (r ^2^ 0.78, p < 0.0001)

Identifying oneself as a faculty member did not predict the PageRank. The participants that identified themselves as either a trainee or organization did not have higher PageRanks. The model predicted a lower PageRank for those participants who failed to identify themselves or whose identity could not be discerned from their Twitter profile ( Table 2).

**Table 2.** Parameter estimates for multivariate linear regression model.

Parameter	β	Standard Error	p
Intercept	0.51	0.078	< 0.0001
Category = faculty	0.068	0.12	0.56
Category = organization	0.27	0.15	0.06
Category = other/cannot categorize	-0.25	0.09	0.005
Number of tweets authored	0.061	0.0063	< 0.0001
Number of tweets re-tweeted	0.067	0.016	< 0.0001

## Discussion

The two main findings in this investigation are: 1) being an experienced source of medical information has no effect on influence within a Twitter learning community and 2) a large percentage of participants do not provide enough information for one to assess their level of experience.

Twitter learning communities are becoming increasingly popular. Both the American Societies of Clinical Oncology and Nephrology (ASCO and ASN, respectively) have begun yearly Twitter learning communities to accompany their annual scientific meetings ^4, 5^. These communities bring together participants of various levels and, effectively, allow each participant to assume the role of both learner and teacher. While “learners” are exposed to a number of “teachers” in these communities, not all participants who assume the role of “teacher” are qualified to do so. Teachers are traditionally considered to have experience regarding the subject matter they teach. These features allow teachers to exert influence over the learners. In our investigation, the most experienced sources of medical information (faculty) exerted the greatest influence in the Twitter community. However, they did so because they had the greatest Twitter activity and not because of their status as faculty members.

Influence that depends only on Twitter activity and not the experience of the composer of a tweet is concerning. Any participant, regardless of his/her experience, could exert a great influence over the community simply by authoring the most tweets. As a result, learners may be receiving medical information from sources of questionable experience. To our knowledge, there has been no literature to support the idea that participants with the greatest Twitter activity are necessarily the most experienced sources of knowledge.

The second and equally concerning finding is the ambiguity in Twitter profiles of a large percentage of participants. Uncategorized participants accounted for 58% of all participants in the #APDIM13 community. While over 91% of Twitter users choose to make their profiles publicly visible, fewer seemed to identify their geographic location (75.3%) or place of origin (71.8%) ^1^. Even fewer choose to identify their gender/sex (64.2%) ^1^. Ambiguity in one’s professional status poses a unique challenge in the medical community. Currently, physicians who use Twitter face an “identity dilemma”, which results in incomplete, inaccurate, and often ambiguous Twitter profiles ^19^. Such profiles make it hard for the learner to assess the experience of the participant dispensing information. Moreover, ambiguous profiles are antithetical to the American Medical Association’s (AMA) principles of medical ethics ^20^. Both the AMA and Twitter-savvy physicians advocate “ownership of activity” in social media by avoiding anonymity and accurately stating one’s credentials ^20, 21^. Given that we could not identify over half of participants in the #APDIM13 Twitter learning community, it is possible that the current regression model is unable to reveal the predictive power of one’s identity on social media influence.

Two limitations deserve a special mention. First, we were unsuccessful at identifying those individuals whose Twitter profiles were ambiguous. In this investigation, we classified them as “other” because the vague Twitter profiles did not allow us to identify them with reasonable certainty. Second, we could not include additional variables into our prediction model. The ambiguous profiles did not include information about age, gender, and/or location. Had we included these variables into our multivariate linear equation, we would have produced an unreliable prediction model.

The greatest strength of this investigation is the method used to measure social media influence. We quantified influence using the number and directionality of mentions within the learning community. Previous studies have used tie strength to measure influence ^22^. Unlike our method, tie strength changes over time, thereby making it difficult to assess one’s influence within a specific learning community. We also used the PageRank algorithm to quantify social media influence. PageRank is considered to be an accurate measurement of influence within social media networks and offers more insight into a person’s influence than simply counting his/her number of followers ^8– 14, 23^.

## Conclusions

**Figure 4.**
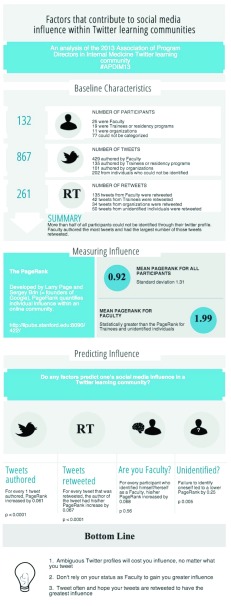
Summary infograph of data.

As the number of Twitter learning communities grow in number and variety, less emphasis will be placed on using social media to exchange medical knowledge. Rather, a greater focus should and will be made towards how to create communities where experienced teachers can 1) be easily identified and 2) have the greatest influence over learners. We must train students to correctly identify experienced sources of information and train those sources to create clear, unambiguous Twitter profiles to allow for easy identification by students. Until then, individuals who consider themselves as experienced educators must actively use Twitter in order to have the greatest influence on learners ( Figure 4).

## Data availability

The data can be downloaded from the Healthcare Hashtag Project website using the hashtag #APDIM13.
