# Supplementary material for: Factors that contribute to social media influence within an Internal Medicine Twitter learning community
Source: F1000Res. 2014 May 29;3:120. [Version 1] doi: 10.12688/f1000research.4283.1 (PMC4111123; doi:10.12688/f1000research.4283.1)
Supplement: Peer review history

# Peer review: Referee response for version 1

**DOI:** 10.5256/f1000research.4585.r4941

**Published:** 2014-06-05

**Authors:** Paul Phelan

**Affiliations:** 1 Department of Nephrology, Beaumont Hospital, Dublin, Ireland

Desai *et al.* have reported on the factors contributing to social media influence in a medical community, specifically regarding Twitter use related to an Internal Medicine conference. With a growing presence of social media and online tools in medical education, this represents a relevant and timely study. They demonstrated that faculty members were more likely to have tweets amplified (retweeted) but faculty were not necessarily more influential (as measure using PageRank). Factors influencing influence were number of tweets i,e  he/she who shouts the loudest, had the most influence. This has implications for how we get our online material and the quality of the source.

Many users (60%) were uncategorized so this undoubtedly took away from the power of the study/conclusions that can be drawn. This is not the fault of the authors however and is a talking point in its own right.

Some minor points:

- Abstract: This portion is confusing to a casual reader: 
*"Faculty had the greatest influence amongst all participants (mean 1.99, p < 0.0001). Being a faculty member had no predictive effect on influence (β = 0.068, p = 0.6)."-*
- Regarding PageRank (which many readers will not be familiar with): Does it have a maximum value or is it indefinite? Are RT's included in PageRank?
- In Results:
*"We identified less than two of every ten participants as a faculty member based on the information from their Twitter profile (19%, 95% CI 13–26%, p < 0.0001)."*
  What does the p value refer to? What is the comparator? This is not immediately obvious to me.
- Figure 3: Does this add any value?

Overall, an interesting & relevant study for medical educators, from authors with a strong track record in this field. It makes us question our sources of online medical material and highlights some issues with twitter use in medical education.

I have read this submission. I believe that I have an appropriate level of expertise to confirm that it is of an acceptable scientific standard.

# Peer review: Referee response for version 1

**DOI:** 10.5256/f1000research.4585.r4940

**Published:** 2014-06-02

**Authors:** Manish Ponda

**Affiliations:** 1 Laboratory of Biochemical Genetics and Metabolism, The Rockefeller University, New York, NY, USA

Social media is an increasingly important tool for medical education.  This article adds to the necessary body of literature for a greater understanding of its applications.

I have read this submission. I believe that I have an appropriate level of expertise to confirm that it is of an acceptable scientific standard.
